# SHIPping out diabetes—Metformin, an old friend among new SHIP2 inhibitors

**DOI:** 10.1111/apha.13349

**Published:** 2019-08-12

**Authors:** Sanna Lehtonen

**Affiliations:** ^1^ Department of Pathology and Research Program for Clinical and Molecular Metabolism, Faculty of Medicine University of Helsinki Helsinki Finland

**Keywords:** diabetes, diabetic kidney disease, insulin resistance, insulin signalling, lipid phosphatase, podocyte

## Abstract

SHIP2 (Src homology 2 domain‐containing inositol 5′‐phosphatase 2) belongs to the family of 5′‐phosphatases. It regulates the phosphoinositide 3‐kinase (PI3K)‐mediated insulin signalling cascade by dephosphorylating the 5′‐position of PtdIns(3,4,5)P3 to generate PtdIns(3,4)P2, suppressing the activity of the pathway. SHIP2 mouse models and genetic studies in human propose that increased expression or activity of SHIP2 contributes to the pathogenesis of the metabolic syndrome, hypertension and type 2 diabetes. This has raised great interest to identify SHIP2 inhibitors that could be used to design new treatments for metabolic diseases. This review summarizes the central mechanisms associated with the development of diabetic kidney disease, including the role of insulin resistance, and then moves on to describe the function of SHIP2 as a regulator of metabolism in mouse models. Finally, the identification of SHIP2 inhibitors and their effects on metabolic processes in vitro and in vivo are outlined. One of the newly identified SHIP2 inhibitors is metformin, the first‐line medication prescribed to patients with type 2 diabetes, further boosting the attraction of SHIP2 as a treatment target to ameliorate metabolic disorders.

## 1 DIABETES AND DIABETIC KIDNEY DISEASE

### 1.1 Diabetes and its complications

Diabetes is a heterogeneous group of diseases characterized by chronic hyperglycemia. It has classically been divided into type 1 (T1D) and type 2 diabetes mellitus (T2D) and a few rare forms of the disease. T1D is characterized by little or no production of insulin by the pancreatic beta‐cells, whereas T2D is characterized by both impaired insulin secretion and insulin resistance, leading to the inability of insulin‐sensitive muscle and adipose tissues to take up glucose. Chronic hyperglycemia leads to damage of blood vessels and consequently, diabetes is associated with both macrovascular and microvascular complications that are major causes for morbidity and mortality.^1^ The macrovascular complications include coronary artery disease, peripheral arterial disease and stroke, and the microvascular complications include diabetic kidney disease, neuropathy and retinopathy.^1^

### 1.2 Diabetic kidney disease

This review concentrates on diabetic kidney disease (DKD), the potentially life‐threatening complication of diabetes and the most common cause of chronic kidney disease in the developed world. As the incidence of diabetes is escalating, also the number of patients suffering from DKD is increasing.^2^ DKD is characterized by gradual increase in albumin excretion into urine, a decrease in glomerular filtration rate, elevated arterial blood pressure and development of glomerulosclerosis. It develops to 20%‐40% of patients with T1D and to even up to 50% of patients with T2D.^3^ The development of DKD is multifactorial involving hyperglycemia‐induced metabolic and hemodynamic processes that are further influenced by genetic and environmental factors. The non‐modifiable risk factors include genetics, sex, age at onset and duration of diabetes.^3^ Also a number of modifiable factors, including glycemic control, blood pressure, lipid abnormalities, smoking, chronic low‐grade inflammation, advanced glycation end products and lack of physical activity, affect the development of DKD.^3^

The pathophysiological changes in the kidney upon development of DKD involve both the tubular and glomerular compartments as well as the vasculature. An early feature of DKD is kidney hypertrophy, occurring in both glomerular and tubular compartments.^4^ The greatest hypertrophic change is observed in the proximal tubules. The growth of the tubules and enhanced Na^+^‐glucose cotransport lead to increased proximal tubule reabsorption and an increase in the glomerular filtration rate through tubuloglomerular feedback.^4^ Increased exposure to different diabetes‐associated metabolites may trigger various pathological pathways associated with tubulointerstitial fibrosis, inflammation, hypoxia and apoptosis that may contribute to the progression of DKD.^4^

### 1.3 Changes in the glomerular filtration barrier in DKD

**Figure 1:**
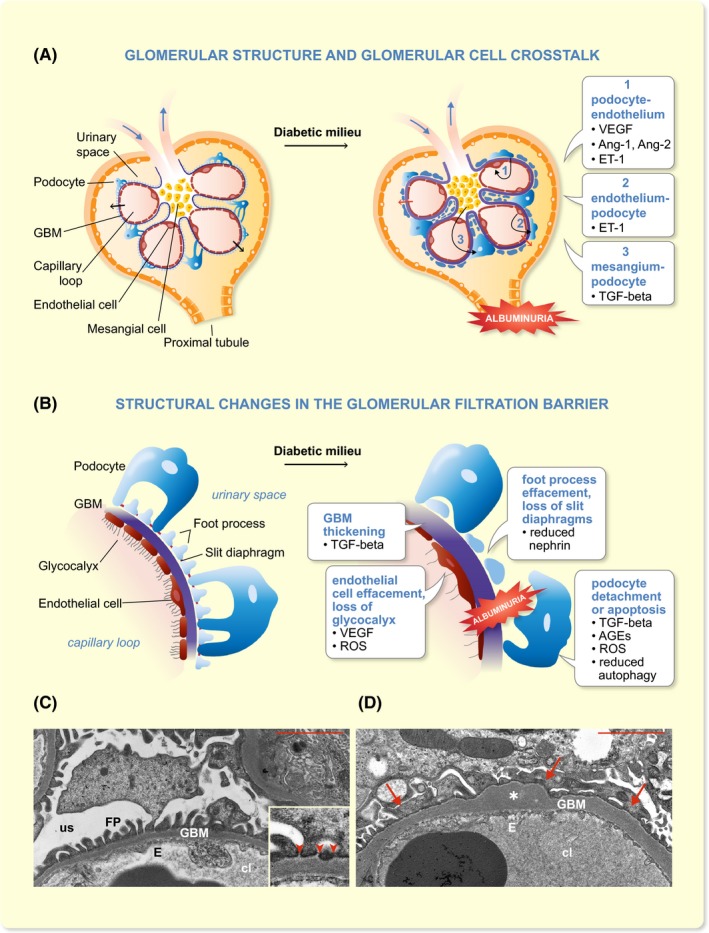
Glomerular filtration barrier and changes observed in diabetic milieu. A, A schematic cartoon of the glomerulus in health and in diabetic milieu. The cartoon also depicts the crosstalk (black, curved arrows in diabetic milieu) between podocytes and endothelium (1), endothelium and podocytes (2) and mesangial cells and podocytes (3). Ang‐1/2, angiopoietin‐1/2; ET‐1, endothelin‐1; GBM, glomerular basement membrane; TGF‐beta, transforming growth factor beta; VEGF, vascular endothelial growth factor. The black arrows indicate size‐ and charge‐selective filtration of plasma through the glomerular filtration barrier in health. Red arrows indicate leakage of albumin (albuminuria) through the damaged glomerular filtration barrier in diabetic milieu. B, A schematic cartoon of the glomerular filtration barrier and factors associated with the indicated changes in diabetic kidney disease. The glomerular filtration barrier consists of three layers, the endothelial cells with glycocalyx, the glomerular basement membrane (GBM) and the podocytes, with their foot processes interconnected with slit diaphragms. In diabetic milieu, the glomerular filtration barrier shows distinct changes associated with the development of albuminuria. These changes include glomerular basement membrane thickening, podocyte foot process and endothelial cell effacement, loss of slit diaphragms and endothelial glycocalyx, and detachment or apoptosis of podocytes. Examples of the factors involved in these changes are indicated. AGEs, advanced glycation end‐products; ROS, reactive oxygen species. C‐D, Electron microscopic images visualizing the glomerular filtration barrier in health and in diabetic kidney disease. C, An electron micrograph of the glomerular filtration barrier in a healthy mouse. The inset shows a higher magnification of the filtration barrier. Arrowheads indicate slit diaphragms. D, An electron micrograph of the glomerular filtration barrier in a mouse model of diabetes (E1‐DN mouse; the image is similar to fig. 6b,c in^21^), showing irregular thickening and bulging of the GBM and foot process effacement. cl, capillary loop; E, endothelial cell; FP, foot process; GBM, glomerular basement membrane; us; urinary space; asterisk, GBM thickening and bulging; arrow, podocyte foot process effacement; arrowhead, slit diaphragm. Scale bar in C and D, 2 µM

Glomerulus is the central site of kidney injury in diabetes. Podocytes, the highly specialized epithelial cells, form together with the fenestrated endothelial cells and the glomerular basement membrane (GBM) the glomerular filtration barrier that prevents the passage of proteins of the size of albumin and larger from the capillary lumen to the urinary space while allowing water and small molecules to freely pass (Figure 1A). Podocytes wrap around the capillaries, with their large cell bodies extending to the urinary space, and their foot processes attaching to the underlying GBM. The neighbouring podocyte foot processes are interconnected by special cell adhesion structures called slit diaphragms (Figure 1B,C), composed of a set of transmembrane proteins and cytosolic adapter molecules linking the slit diaphragm to the underlying actin cytoskeleton and signalling networks.^5^ One of the central molecules of the slit diaphragm is the immunoglobulin superfamily member nephrin, shown to be essential for an intact glomerular filtration barrier as mutations in it lead to the congenital nephrotic syndrome of the Finnish type.^6^ The compartmentalization of the apical and basolateral domains of the podocytes by the slit diaphragm allows the expression of distinct sets of proteins in a polarized manner. As an example, podocalyxin is expressed in the apical domain of podocytes and with its negative charge, created by a glycocalyx, apparently maintains the glomerular filtration barrier open.^7^ More detailed molecular characterizations of podocytes and the slit diaphragm are provided in recent reviews.^5^, ^8^

The diabetic milieu, exposing glomerular cells to high glucose, fatty acids, growth factors, cytokines and hormones, leads to glomerular hypertrophy and distinct changes in the structure and function of the glomerular filtration apparatus (Figure 1A,B). Histologically, DKD is characterized by mesangial matrix expansion and eventually glomerulosclerosis. At the electron microscopic level, the first sign of DKD is the thickening of the GBM. This is followed by effacement of the podocyte foot processes, loss of slit diaphragms and loss of podocytes by detachment or apoptosis, and progressive albuminuria (Figure 1B‐D).^9^ Podocytes, endothelial cells and mesangial cells are engaged in multidirectional crosstalk (illustrated in Figure 1A), and this has been shown to affect the development and progression of DKD, involving a number of pathways, as summarized below.

### 1.4 Podocyte‐mesangial cell axis in DKD

A central contributor to glomerular damage is hyperglycemia‐induced activation of the intrarenal renin‐angiotensin‐aldosterone system (RAAS) and production of excess angiotensin II.^10^ Angiotensin II can damage podocytes, endothelial cells and mesangial cells by activating pathways associated with extracellular matrix accumulation, fibrosis, inflammation and generation of reactive oxygen species (ROS).^10^ Increased generation of intracellular ROS leads to podocyte apoptosis.^11^ Podocyte loss is deleterious as podocytes are terminally differentiated cells and they lack the capacity to proliferate. The absolute number of podocytes can predict the progressive decline in renal function and proteinuria^12^; when podocyte loss exceeds 40%, sustained high‐grade proteinuria ensues and renal function declines.^13^

A central regulator of the synthesis of extracellular matrix proteins is transforming growth factor beta (TGF‐β), produced by mesangial cells.^14^ TGF‐β‐induced stimuli contribute to glomerular basement membrane thickening, mesangial matrix production and loss of podocytes.^14^ TGF‐β‐ induced podocyte loss may occur *via* apoptosis^15^ caused by activation of the mammalian target of rapamycin (mTOR) pathway, stimulation of mitochondrial oxidative phosphorylation and generation of ROS.^16^ TGF‐β may also induce detachment of viable podocytes *via* epithelium‐to‐mesenchyme transition (EMT),^17^ that is, by inducing downregulation of epithelial and upregulation of mesenchymal proteins, which may lead to podocyte detachment.^17^ Podocyte apoptosis may also be caused by hyperglycemia‐induced generation of advanced glycation end products (AGEs), which induce generation of intracellular ROS.^18^ Also reduction of autophagy, the lysosomal degradation pathway that maintains cellular homoeostasis, leads to podocyte apoptosis.^19^, ^20^ Of the slit diaphragm proteins, nephrin, for example, has been shown to be either downregulated^21^, ^22^ or mislocalized^23^, ^24^ in animal models of DKD, and the apical domain protein podocalyxin has been shown to be downregulated in the kidneys of diabetic rats.^25^ These changes contribute to podocyte foot process effacement and loss of slit diaphragms. As visualized by the above examples, a multitude of mechanisms contribute to podocyte injury and dysfunction (summarized in Figure 1B), as described in more detail in recent reviews.^26^, ^27^

### 1.5 Endothelial cell‐podocyte axis in DKD

Abnormal angiogenesis and endothelial cell damage in glomeruli are also central contributors to the development of DKD. New blood vessel formation has been observed in both T1D and T2D, and this contributes to glomerular hypertrophy in the early stages of diabetic kidney injury.^28^, ^29^ Morphological studies have revealed a reduction in glomerular capillary endothelial fenestrations and damage of the endothelial glycocalyx (Figure 1B).^30^, ^31^, ^32^ The proangiogenic factor vascular endothelial growth factor (VEGF) plays a key role in abnormal angiogenesis in the glomeruli in diabetes, and its expression correlates with new blood vessel formation in glomeruli.^33^ VEGF isoforms VEGFA and VEGFC are both produced by podocytes and their receptors are expressed in endothelial cells.^34^, ^35^ The expression of both VEGFA and its receptor, VEGF receptor 2, is increased in experimental diabetes.^36^ VEGFA is associated with glomerular endothelial cell damage and vascular dysfunction but interestingly, various VEGFA isoforms confer differing effects on the kidney.^37^ In line with this, a recent study revealed that upregulation of VEGFA_165_b isoform in mouse podocytes in vivo protects against diabetic kidney injury and albuminuria by phosphorylating VEGF receptor 2 in endothelial cells and restoring the endothelial glycocalyx damaged by diabetes.^37^ In experimental diabetes, VEGFC enhances the synthesis of endothelial glycocalyx and reduces albumin permeability in glomeruli, and thereby reduces the development of diabetic kidney injury.^38^ While animal experiments have suggested promise for antiangiogenic therapies, and especially for anti‐VEGF antibodies for treating DKD, findings in human, demonstrating development of renal injury and proteinuria as summarized in a recent review,^39^ have raised a concern about blocking VEGF signalling. Concomitantly, it appears that either too much or too little of VEGFA can lead to pathological changes in the kidney.^39^ Interestingly, inhibition of VEGFB signalling in experimental DKD models appears to protect against DKD—*via* non‐angiogenic mechanisms—by ameliorating renal lipotoxicity and thereby reducing pathological changes in the kidney, sensitizing podocytes to insulin and preventing renal dysfunction.^40^

The actions of VEGF are modulated by angiopoietin‐1, which is expressed in podocytes, while its receptor Tie2 is found in glomerular endothelium.^41^ In experimental diabetes, angiopoietin‐1 level is decreased concomitant to increased VEGFA and signs of diabetic kidney injury, including aberrant angiogenesis.^42^ Some of these changes, including proliferation of endothelial cells and albuminuria, could be reverted by targeted overexpression of angiopoietin‐1 in podocytes.^42^ Furthermore, the expression of angiopoietin‐2, a natural antagonist of angiopoietin‐1 signalling,^43^ was shown to be upregulated in podocytes and glomerular endothelial cells in an experimental model of diabetes concomitant with low expression of angiopoietin‐1.^44^ As podocyte‐specific overexpression of angiopoietin‐2 causes apoptosis of endothelial cells,^45^ the reduced angiopoietin‐1/angiopoietin‐2 ratio may be a contributing factor in the development of DKD.^44^ A recent study revealed increased mitochondrial dysfunction specifically in glomerular endothelial cells, but not in podocytes, in experimental streptozotocin‐induced diabetes in a mouse strain susceptible to diabetic kidney injury.^46^ The endothelial cells of these mice showed signs of injury and loss of fenestrae at an early stage of the disease. The mice also showed podocyte loss and albuminuria, and increased expression of endothelin‐1 receptor type A in the glomeruli, and increased circulating endothelin‐1.^46^ Notably, reducing mitochondrial stress pharmacologically or blocking endothelin‐1 receptor type A reduced injury of the endothelial cells and ameliorated podocyte loss and albuminuria.^46^

Crosstalk between the different cell types is also essential in the tubulointerstitial compartment and may play an important role in renal disease progression.^47^ More detailed discussion on the crosstalk between the different renal cell types is beyond the scope of this review, but the given examples underscore the complexity of the regulation of the glomerular filtration and emphasize the role of all glomerular cell types in maintaining the normal kidney function, as well as in contributing to the dysfunction of the filtration barrier in renal diseases, including DKD. The topic of glomerular cell crosstalk has been extensively covered by several recent reviews.^47^, ^48^, ^49^, ^50^

### 1.6 Mitochondrial dysfunction and hypoxia in DKD

The function of mitochondria and health of kidney are closely interlinked due to high energy and oxygen demands of the kidney, and thereby mitochondrial dysfunction is considered as a central contributor to the development and progression of DKD.^51^, ^52^, ^53^ Mitochondria regulate the levels of reactive oxygen species (ROS), cytosolic calcium and apoptosis and produce ATP for basic cellular functions as well as for cellular repair. An experimental model of diabetes in rat revealed decreased ATP content and fragmentation of mitochondria in proximal tubule cells prior to the onset of proteinuria, supporting a role for mitochondrial dysfunction as a driver in the development of DKD.^54^ While low levels of ROS, such as superoxide anions, are beneficial for cellular functions, excessive generation of ROS is a general sign of mitochondrial dysfunction in the kidney and toxic to the mitochondria and the cells.^51^, ^52^ Oxidative stress contributes to increased apoptosis and fibrosis, leading to declined renal function. A recent study revealed that inhibition of apoptosis signal‐regulating kinase 1 (ASK1), which induces apoptotic, fibrotic and inflammatory signalling in states of oxidative stress, reduces inflammation and fibrosis and prevents the reduction of the glomerular filtration rate in rodent models of DKD.^55^ This supports the hypothesis of beneficial effects of reducing oxidative stress in DKD. Another recent study searched for factors that protect from DKD and found that pyruvate kinase M2 (PKM2), a glycolytic enzyme, was elevated in patients with over 50 years duration of diabetes without signs of DKD.^56^ Activation of PKM2 in rodent models of diabetes normalized metabolic parameters and mitochondrial dysfunction and ameliorated kidney pathology, suggesting that increasing the glycolytic flux and mitochondrial biogenesis protects glomeruli from the toxicity caused by hyperglycemia.^56^ Mitochondrial function has also been linked with insulin signalling.^57^ The researchers showed that podocyte‐specific depletion of mitochondrial fusion‐associated protein prohibitin‐2 (PHB2) in mice results in proteinuria, renal failure and death of the animals.^57^ Strikingly, however, depletion of PHB2 in mice and cultured podocytes indicated that despite of the morphological changes of the mitochondria, the oxidative phosphorylation system was not impaired and also the oxygen consumption of podocytes lacking PHB2 remained normal. However, depletion of PHB2 resulted in insulin receptor‐mediated hyperactivity of mTOR signalling. In line with this, knocking out insulin receptor either alone or in combination with IGF‐1 receptor in mice depleted of PHB2 delayed the onset of renal failure without affecting the mitochondrial phenotype. The triple knockout animals remained proteinuric, though, indicating that an mTOR‐independent pathway regulates the proteinuria.^57^ Nevertheless, this study provides compelling evidence interconnecting mitochondrial dysfunction and insulin signalling, and emphasizes the importance of maintaining the activity of the insulin signalling cascade at an appropriate level.

Mitochondrial function and ROS production together with increased oxygen consumption are intimately linked to tissue hypoxia.^53^, ^58^ In diabetes, hyperglycemia increases the oxygen demand of the kidney and when not matched with increased oxygen delivery, intrarenal hypoxia emerges.^59^ Concomitantly, Franzén *et al*.^60^ measured intrarenal oxygen tension in diabetic mice, revealing that kidney hypoxia precedes albuminuria. This suggests that improving oxygen homoeostasis in the kidney could provide a way to treat DKD. Of note, however, specific targets may have highly cell‐specific consequences. Activating, for example, hypoxia inducible factor (HIF) in kidney tubules is beneficial for mitochondrial function and protects the kidney, whereas HIF activation in glomeruli leads to glomerulosclerosis and proteinuria.^58^ Altogether, the maintenance of mitochondrial homoeostasis, that is, mitochondrial biogenesis, fission and fusion as well as elimination of damaged, non‐functional mitochondria, is essential for maintaining normal kidney function.^51^ Studies have revealed that several antidiabetic drugs affect various mitochondrial functions independent of their hypoglycemic effects, including both protective and harmful effects, as highlighted by a recent review.^61^

### 1.7 Endoplasmic reticulum stress in DKD

Various diabetes‐associated factors, including hyperglycemia, proteinuria and increased levels of free fatty acids and advanced glycosylation end products, contribute to the development of endoplasmic reticulum (ER) stress that advances the progression of DKD.^62^, ^63^ ER stress markers have been shown to be upregulated in the kidneys of experimental animals as well as of patients with T2D.^63^ In DKD, protein misfolding and ER stress are evident in both podocytes and the tubular compartment, and can lead to apoptosis and extracellular matrix production.^62^ The 78‐kDa glucose‐regulated protein (GRP78) is a classical ER chaperone and a central regulator of protein folding processes, and, thus, it plays a key role in maintaining protein homoeostasis.^64^ A recent study revealed that high glucose treatment induces translocation of GRP78 to the plasma membrane in mesangial cells, where it binds to integrin β1, activates focal adhesion kinase and the downstream PI3K/AKT signalling.^64^ This enhances the synthesis of extracellular matrix proteins and glomerular fibrosis.^64^ Also other studies have linked insulin signalling to ER stress in diabetes. Madhusudhan *et al*.^65^ found that impaired insulin signalling together with hyperglycemia prevents the nuclear translocation of transcription factor spliced X‐box binding protein 1 (sXBP1), which resulted in maladaptive ER stress signalling in podocytes and worsened DKD. Concomitantly, the same team found that coagulation protease activated protein C (aPC) maintains ER homoeostasis *via* sXBP1 in podocytes.^66^ Specifically, they revealed that aPC can compensate for the absence of insulin receptor in podocytes in mice with experimental diabetes. This occurred by activation of the PI3K p85‐subunit‐dependent nuclear translocation of sXBP1, which prevented the maladaptive ER stress.^66^

### 1.8 Epigenetic changes in DKD

In addition to transcription factors and classical signalling cascades, genes associated with DKD are regulated by epigenetic mechanisms.^67^ Epigenetic changes do not alter the underlying DNA sequence but affect the accessibility of chromatin and expression of genes *via* DNA methylation, chromatin histone modifications and non‐coding RNAs. Environmental factors play a key role in the development of DKD, and the epigenetic mechanisms mediate the crosstalk between the genes and the environment. Epigenetic changes are also apparently involved in the so called metabolic memory, in which the gene expression pattern induced by high glucose persists even though the glycemic control has been achieved.^67^ The emerging developments in the epigenetic research in DKD may open new therapy options for DKD. A recent study revealed increased expression of DNA methyltransferase 1 in peripheral blood mononuclear cells in patients with DKD.^68^ This altered the methylation of the promoter regions of the regulators of the mTOR signalling pathway, leading to activation of the pathway and increased inflammation in the diabetic kidneys.^68^ Majumder *et al*.^69^ showed that modifying histone methylation in podocytes affects the course of glomerular diseases. They found that histone H3 lysine 27 trimethylation (H3K27me3) is diminished and the expression of demethylase UTX is increased in patients with DKD. This induces dedifferentiation of podocytes and accelerates the development of glomerular injury. Concomitantly, inhibiting the demethylase in diabetic *db/db* mice ameliorated kidney disease, further supporting the hypothesis that targeting the epigenetic events may be beneficial in treating DKD.^69^

### 1.9 Insulin signalling in DKD

Understanding the complexity of the molecular and cellular interaction networks and the exact molecular mechanisms involved in renal cell crosstalk will aid in designing new intervention strategies to slow down or stop the progression of renal diseases. At the same time, it has become apparent that the heterogeneous nature of diabetes makes treating it challenging, and the available treatment strategies often fail to stop the progression of the disease and its complications. Many of the studies above point out the potential of targeting a specific molecule or certain cellular modifications to prevent the development of DKD, yet we lack the personalized treatment options, called for due to the complex nature of the disease. A recent study describes a data‐driven cluster analysis of six variables in patients with newly characterized diabetes. The authors identified five clusters of patients with diabetes, each subgroup specified by distinct disease progression and risk of diabetic complications. Interestingly, one of the clusters, the patients most resistant to insulin, showed a high risk to develop DKD.^70^ In addition, previous studies have revealed that insulin resistance is associated with microalbuminuria in patients with either T1D^71^ or T2D^72^, ^73^ as well as in healthy people without diabetes,^74^ and that insulin resistance precedes and predicts the development of both microalbuminuria and nephropathy in T1D.^75^, ^76^ These data highlight the importance of insulin signalling in maintaining normal kidney function and insulin resistance as a central contributor to the development of kidney disease and albuminuria. It is also interesting to note that several of the mechanisms that participate in driving the development of DKD, as presented in the paragraphs above, converge with the insulin signalling pathway, further underlining the importance of this pathway as a central contributor to kidney health.

## 2 DEFECTS IN INSULIN SIGNALLING AND GLUCOSE TRANSPORTER TRAFFICKING LEAD TO THE DEVELOPMENT OF INSULIN RESISTANCE

**Figure 2:**
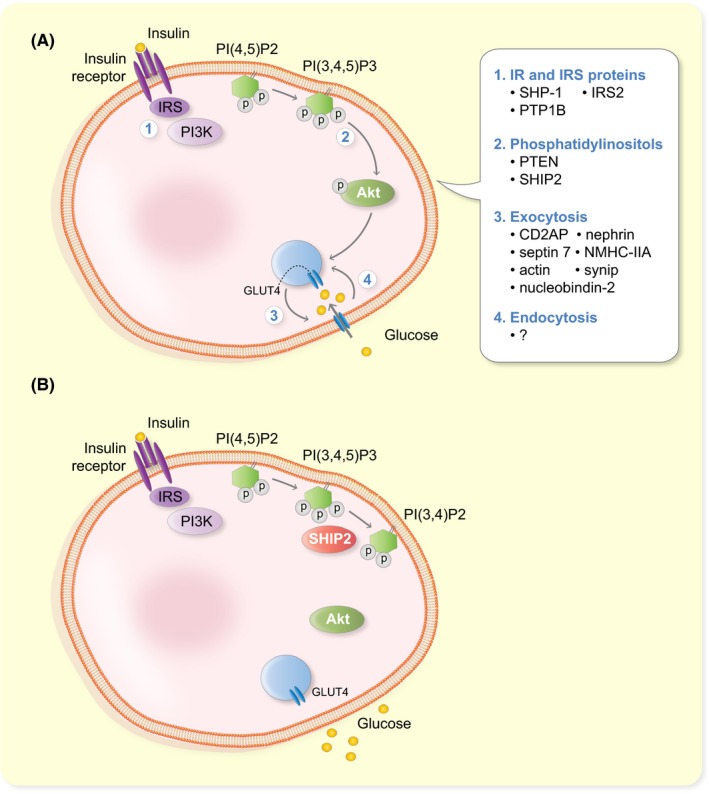
SHIP2 regulates the insulin signalling pathway. A, Simplified cartoon of the insulin signalling pathway indicating various points of regulation of its activity. Binding of insulin to its receptor leads to activation of phosphatidylinositol 3‐kinase (PI3K), phosphorylation of phosphatidylinositol 4,5‐bisphosphate (PI(4,5)P2) to phosphatidylinositol 3,4,5‐trisphosphate (PI(3,4,5)P3) and subsequent activation (phosphorylation, P) of Akt. This leads to translocation of insulin responsive glucose transporter 4 (GLUT4) to the plasma membrane and uptake of glucose into cells. The activity of the pathway can be modulated at the level of (1) the insulin receptor and insulin receptor substrate (IRS) proteins, (2) phosphorylation/dephosphorylation of phosphatidylinositols and (3) exocytosis or (4) endocytosis of GLUT4. Examples of proteins regulating the different steps in podocytes are indicated. B, SHIP2 suppresses the insulin signalling pathway by hydrolysing PI(3,4,5)P3 to PI(3,4)P2. This reduces glucose uptake

Understanding the mechanisms leading to the development of insulin resistance is essential as insulin resistance is a key factor in the pathogenesis of diabetes and DKD, as described above.^70^, ^77^ Development of insulin resistance is caused by either reduced activity of the insulin signalling cascade or impaired trafficking of the insulin responsive glucose transporter 4 (GLUT4) to the plasma membrane.^78^ Insulin signalling is initiated by binding of insulin to insulin receptor leading to its activation, the consequent phosphorylation of a number of downstream substrates and activation of the phosphatidylinositol 3‐kinase (PI3K) pathway (Figure 2A). PI3K phosphorylates phosphatidylinositol 4,5‐bisphosphate (PtdIns(4,5)P2) to phosphatidylinositol 3,4,5‐trisphosphate (PtdIns(3,4,5)P3), which is a key lipid second messenger in various metabolic effects of insulin. To enhance glucose uptake, PtdIns(3,4,5)P3 transfers signals to downstream molecules including serine/threonine kinase Akt.^78^ In the basal state, GLUT4 resides mainly in the cytoplasm and only a small proportion is detected at the plasma membrane, but insulin‐induced activation of Akt induces rapid translocation of GLUT4‐containing vesicles to the cell surface.^79^, ^80^ The trafficking is controlled at various steps with distinct sets of proteins. Specifically, the GLUT4‐containing vesicles contain vesicle‐SNAREs (soluble N‐ethylmaleimide‐sensitive factor attachment protein receptors), which interact with target‐SNAREs on the plasma membrane. These together, further aided by sets of accessory proteins, facilitate the translocation, docking and fusion of the GLUT4‐containing vesicles with the plasma membrane leading to uptake of glucose into cells (Figure 2A).^79^, ^80^

Defects either in the insulin receptor itself, for example, changes in its expression level or activity, in its downstream substrates or molecular regulators of the signalling cascade may lead to impaired activation of the insulin signalling pathway and insulin resistance (Figure 2A).^78^ However, the numbers of individuals with genetic defects in the insulin receptor are rare, proposing that peripheral insulin resistance is rather caused by defects downstream of the insulin receptor. It is plausible that the cause of insulin resistance is multifactorial, a combination of genetic defects and environmental factors, or that it is caused by simultaneous lower functional input of multiple components of the pathway, which in the end is insufficient to lead to full activation of the signalling cascade and uptake of glucose into cells.^78^ The complex regulation of the trafficking of GLUT4‐containing vesicles *via* various accessory molecules at different steps makes also the trafficking process a potential site for alterations, which may lead to impaired translocation of GLUT4 to the plasma membrane and consequent insulin resistance (Figure 2A).^79^, ^80^

## 3 INSULIN RESISTANCE AND DIABETIC KIDNEY DISEASE

### 3.1 Insulin resistance of podocytes and DKD

The seminal study by *Ahlqvist et al*.^70^ shows that peripheral insulin resistance plays a central role in the development of DKD, but it has remained open whether insulin resistance of podocytes could directly contribute to the disease progression. Studies in animal models of diabetes and DKD, supplemented by studies on cultured podocytes, have revealed that podocytes can develop insulin resistance. Podocytes express all the central components of the insulin signalling pathway as well as glucose transporters GLUT4 and GLUT1, both shown to be insulin responsive in podocytes, and can increase their glucose uptake upon insulin stimulation.^81^ Subsequent studies revealed that podocytes isolated from diabetic *db/db* mice show reduced level of Akt activation compared to their lean controls. Also podocytes isolated from the *db/db* mice, before the mice had developed albuminuria, were unable to phosphorylate Akt in response to insulin stimulation.^82^ The indication that insulin resistance of podocytes drives kidney dysfunction was provided by a study revealing that knocking out insulin receptor specifically in podocytes in mice leads to the development of DKD even under normoglycaemia.^83^ These mice showed typical structural features of DKD, including thickening of the GBM, podocyte foot process effacement, signs of podocyte loss by apoptosis and glomerulosclerosis and started developing albuminuria at 5 weeks of age.^83^ A recent study utilizing cultured podocytes revealed that high concentrations of insulin lead to downregulation of insulin receptor *via* proteasome‐dependent lysosomal degradation pathway, and this leads to insulin resistance of podocytes.^84^

### 3.2 Regulation of insulin signalling in podocytes

Several proteins that regulate the activity of the insulin signalling pathway and glucose uptake in podocytes have been identified, and examples of them, and the steps they functionally control, are presented in Figure 2A. At the level of the insulin receptor (Figure 2A, step 1), the protein tyrosine phosphatase Src homology‐2 domain containing phosphatase‐1 (SHP‐1), that is upregulated by high glucose, suppresses insulin signalling by dephosphorylating insulin receptor‐β.^85^ Another regulator of the insulin receptor is protein tyrosine‐phosphatase 1B (PTP1B). Inhibition of PTP1B was shown to have anti‐diabetic effects by improving insulin and leptin signalling in a model of diet‐induced obesity.^86^ Furthermore, knocking down PTP1B activates the PI3K pathway and protects podocytes from endoplasmic reticulum (ER) stress, which associates with podocyte apoptosis and proteinuria in DKD.^87^ Activated insulin receptor phosphorylates its downstream mediators insulin receptor substrates 1 and 2 (IRS1, IRS2). In podocytes, insulin activates IRS2 rather than IRS1.^88^ *Irs2*‐depleted podocytes show insulin resistance, which is caused by upregulation of a protein called phosphatase and tensin homolog deleted on chromosome 10 (PTEN). PTEN dephosphorylates the 3′‐position of PtdIns(3,4,5)P3 to generate PtdIns(4,5)P2, and consequently, upregulation of PTEN diminishes phosphorylation of Akt and reduces the activity of the insulin signalling pathway (Figure 2A, step 2). Knockdown of PTEN leads to phosphorylation of Akt and thus activation of the pathway. However, instead of protecting from ER stress, as in the case of PTP1B, this sensitizes podocytes to ER stress and apoptosis reflecting the complexity of the regulation of ER stress in podocytes.^87^ Another lipid phosphatase that regulates insulin signalling at the level of phosphoinositides (Figure 2A, step 2) is Src homology 2 domain‐containing inositol 5′‐phosphatase 2 (SHIP2). It acts, similarly as PTEN, on PtdIns(3,4,5)P3, but hydolyses its 5′‐position generating PtdIns(3,4)P2 and thereby activates the insulin signalling pathway.^89^ The role of SHIP2 as a suppressor of insulin signalling is described in more detail in paragraph 4.

### 3.3 Regulation of glucose transporter trafficking in podocytes

Insulin‐stimulated glucose uptake can be regulated either during the exocytosis of the GLUT4 containing vesicles (GCVs) from the cytoplasmic pool to the plasma membrane (Figure 2A, step 3) or during their endocytosis (Figure 2A, step 4) to the endosomal compartment for recycling. For exocytosis, GLUT4 needs to be sorted first into GCVs, and this is controlled by adapter protein CD2‐associated protein (CD2AP) in podocytes.^90^ When CD2AP is missing, GLUT4 remains as perinuclear clusters, and insulin‐stimulated glucose uptake is reduced.^90^ Translocation of GCVs to the plasma membrane requires intact actin cytoskeleton, and in podocytes, insulin stimulates reorganization of the cortical actin cytoskeleton in an insulin receptor‐dependent manner.^83^ The docking and fusion of GCVs with the plasma membrane is facilitated by nephrin, which associates with the GCV‐protein vesicle‐associated membrane protein 2 (VAMP‐2).^91^ Nephrin also associates with the small cytoskeletal GTPase septin 7, and the protein complex containing septin 7 plays a central role in controlling the vesicle docking and fusion.^92^, ^93^, ^94^ It has been proposed that septin 7 forms a filamentous barrier to hinder GCV translocation to the plasma membrane thereby reducing glucose uptake.^93^ Depletion of septin 7 enhances the interaction between nephrin and VAMP2 to facilitate the docking and fusion of GCVs with the plasma membrane, leading to increased glucose uptake.^93^ In addition to nephrin, also non‐muscle myosin heavy chain IIA (NMHC‐IIA) and the membrane protein SNAP23 are found in the septin 7 complex and, interestingly, septin 7 regulates the activity of non‐muscle myosin IIA in this complex.^92^ Accordingly, depletion of septin 7 leads to enhanced activity of non‐muscle myosin IIA in the complex, and this increases GCV docking and fusion and uptake of glucose into cells.^92^ Knockdown of NMHC‐IIA or nucleobindin‐2, yet another interaction partner of septin 7, both reduce glucose uptake into podocytes.^92^, ^94^ Also phosphorylation of synip on serine 99 is necessary for GCV translocation and glucose uptake in podocytes.^95^ Thus far, no regulators of GCV endocytosis in podocytes have been identified (Figure 2A, step 4).

### 3.4 Targeting insulin resistance—a way to personalized medication in DKD?

Defining whether podocytes in patients with DKD are insulin resistant is challenging, and the rarity of normoglycaemic patients who show insulin resistance and develop DKD suggest that development of DKD involves a combination of multiple factors in addition to insulin resistance.^96^ Recent reviews summarize in more detail the molecular mechanisms involved in the development of the kidney complication in diabetes, including the role of insulin resistance in the context of the insulin signalling and glucose transporters.^80^, ^97^, ^98^ The significance of insulin signalling in maintaining the kidney function is supported by intervention studies in both patients with diabetes^99^, ^100^, ^101^ and experimental animal models of diabetes^102^, ^103^ showing that insulin sensitizers are renoprotective. These data together support the potential of targeting the components and regulators of the insulin signalling pathway as a strategy for developing new therapeutic interventions to preserve the kidney function in diabetes. Furthermore, the new subclassification of diabetes, identifying insulin resistance as a central risk factor for the development of DKD, allows to better predict the disease progression and outcome, and to design personalized treatments for patients in each subclass, including the severely insulin resistant ones at high risk for DKD.^104^

## 4 SHIP2 SUPPRESSES THE INSULIN SIGNALLING PATHWAY

**Figure 3:**
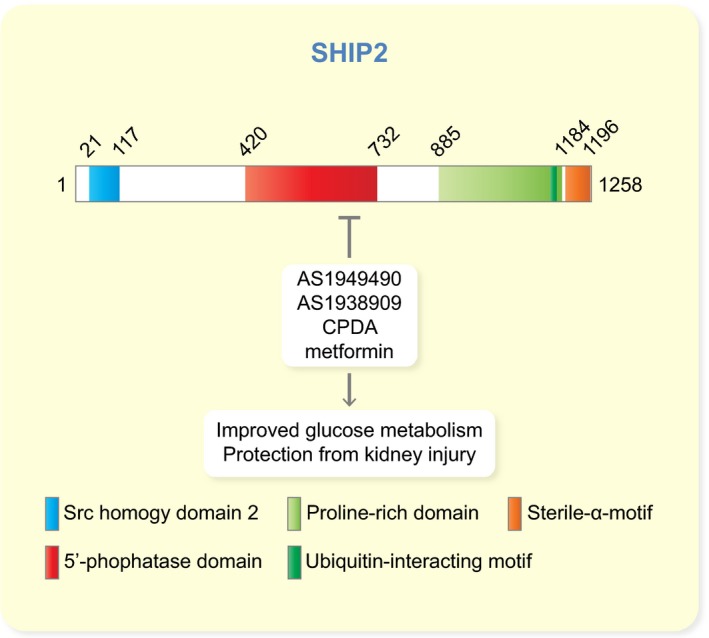
Schematic cartoon of the domain structure of SHIP2 and SHIP2 inhibitors that have been shown to have metabolic effects. The numbers indicate the start and end amino acids of the different domains and motifs

As described above, one of the potential therapeutic target molecules that regulates the insulin signalling pathway is the lipid phosphatase SHIP2 (Figure 2B). SHIP2 is widely expressed in different tissues, with high mRNA expression in human skeletal muscle, heart and placenta.^89^ In mouse, SHIP2 mRNA was detected in all tissues analysed,^105^ and a *LacZ* reporter gene analysis (*LacZ* reporter inserted into the *Inppl1* locus) in the *Inppl1^−/−^* (SHIP2‐depleted) mice revealed high expression of the *LacZ* reporter in brain, skeletal muscle and heart and lower in liver, lung and kidney.^106^ SHIP1, the close homologue of SHIP2 that shares approx. 38% sequence identity with SHIP2, is expressed mainly in cells of hematopoietic origin and during spermatogenesis.^107^ SHIP2 has several conserved domains, including the NH_3_‐terminal Src homology 2 (SH2) domain, the central 5′‐phosphatase domain and the COOH‐terminal proline‐rich and sterile ⍺ motif (SAM) domains (Figure 3). The SH2 domain is missing from the short isoform of SHIP2, generated by alternative splicing. In addition, SHIP2 harbours several tyrosine, serine and threonine phosphorylation sites (reviewed in 108). As SHIP2 suppresses the insulin signalling pathway by dephosphorylating PtdIns(3,4,5)P3 to PtdIns(3,4)P2,^89^ leads elevated expression level or increased activity of SHIP2 to reduced activation of Akt and reduced uptake of glucose into cells (Figure 2B), as shown in various cell culture models.^109^, ^110^, ^111^ It has been proposed that the catalytic activity of SHIP2 is controlled by its tyrosine phosphorylation,^112^, ^113^ but this has remained controversial and, in addition, it has been proposed that serine and threonine phosphorylation could play a role in regulating SHIP2 activity.^114^ The ability of SHIP2 to negatively regulate Akt activation necessitates the translocation of SHIP2 to the plasma membrane, where its central substrate, PtdIns(3,4,5)P3, resides.^115^ In addition to its catalytic phosphatase activity, SHIP2 has other independent functions mediated *via* its numerous interaction partners. Thereby SHIP2 regulates various cellular processes, including PI3K‐mediated insulin signalling, FGF‐ and EGF‐mediated signalling, cell spreading, migration and adhesion, actin cytoskeleton dynamics, endocytosis and apoptosis. Mutations in *INPPL1* (gene encoding SHIP2) have been revealed in opsismodysplasia, a disease of bone maturation,^116^, ^117^ and *via* its various activities, SHIP2 associates with many other diseases, including diabetes, cancer, atherosclerosis and neurodegenerative diseases (for reviews, see, for example 108, 118, 119). This review concentrates on SHIP2 in diabetes.

## 5 GENETIC STUDIES OF SHIP2 IN DIABETES AND DIABETIC KIDNEY DISEASE

SHIP2 is encoded by the *INPPL1* gene located on human chromosome 11.^89^ Several genetic studies link SHIP2 to metabolic disorders, showing that polymorphisms in *INPPL1* may contribute to the pathogenesis of the metabolic syndrome, hypertension and T2D (Table 1). The first piece of evidence was provided by finding a mutation (R1142C) in the proline‐rich region of the protein in the Goto‐Kakizaki rats, a model for T2D, and in spontaneously hypertensive rats, apparently affecting the binding of SH3‐domain containing proteins to this region.^120^ Overexpression of the mutated form of SHIP2 in CHO cells overexpressing the insulin receptor (CHO‐IR) resulted in decreased Akt activation, slightly impairing insulin signalling.^120^ Further work on patients with T2D identified a patient with a 16‐base pair deletion in the 3‐prime untranslated region of *INPPL1*, potentially affecting protein synthesis. Indeed, reporter gene studies with the mutated region indicated that the deletion results in increased mRNA and protein expression, and could thus contribute to increased SHIP2 expression and insulin resistance.^120^ Further analysis of patients with T2D of Caucasian origin in the UK and Belgium revealed a significant association of the 16‐base pair deletion and T2D, proposing that *INPPL1* is a candidate gene contributing to the genetic susceptibility of T2D.^120^ A study in Japanese patients with T2D revealed a number of single nucleotide polymorphisms (SNPs), several of which were located in the 5′‐phosphatase catalytic region of SHIP2. However, many of the SNPs were detected more frequently in the controls than in patients with T2D.^121^ In vitro overexpression studies in CHO‐IR cells revealed that overexpression of a construct including a particular SNP located in the 5′‐phosphatase region inhibited insulin‐induced phosphorylation of Akt2 less potently than overexpression of wild‐type SHIP2, supporting a protective role for this SNP against insulin resistance in this cohort of patients with T2D.^121^ Another study in a Japanese cohort identified SNPs in the promoter and 5‐prime untranslated region of *INPPL1* and investigated their association with impaired fasting glycaemia.^122^ Overexpression of one of the identified SNPs in cultured cells caused an increase in the promoter activity, suggesting that SNPs in this region could at least partly account for impaired fasting glycaemia at least in the patient cohort in question.^122^ SNPs in *INPPL1* have also been found to be associated with the metabolic syndrome. A study with Finnish patients with T1D identified an association of two SNPs in *INPPL1* with the metabolic syndrome in men,^123^ and another study on British patients with T2D found an association of SNPs and haplotypes of *INPPL1* with hypertension and other components of the metabolic syndrome.^124^ The metabolic effects of the mutations and single nucleotide polymorphisms in *INPPL1* are summarized in Table 1.

**Table 1:** Mutations and single nucleotide polymorphisms (SNPs) in the SHIP2 gene *INPPL1* and their metabolic consequences

SNP/mutation	Effect	Species	Reference
R1142C	Slight impairment of insulin signalling	Rat	Marion *et al*.^120^
16‐bp deletion, 3′‐UTR	Increased SHIP2 expression, association with T2D	Human	Marion *et al*.^120^
L632I	Protection against insulin resistance	Human	Kagawa *et al*.^121^
+334 C/T	Impaired fasting glycemia	Human	Ishida *et al*.^122^
rs2276048 rs2276047	Association with the metabolic syndrome in men with T1D	Human	Hyvönen *et al*.^123^
snp8 rs2276047 rs9886	Association with hypertension and other components of the metabolic syndrome	Human	Kaisaki *et al*.^124^

In the above studies, control subjects showed in *INPPL1* SNPs that are protective against T2D. Patients with T2D, on the other hand, had in *INPPL1* SNPs that are associated with and could predispose to the metabolic syndrome, hypertension and T2D. In studies with mice, SHIP2 protein in diabetic *db/db* mice has been shown to be upregulated in insulin‐sensitive tissues, including skeletal muscle, adipose tissue^125^ and podocytes,^126^ apparently contributing to insulin resistance. Interestingly, SHIP2 was upregulated in podocytes prior to the development of albuminuria, and this observation was confirmed in insulin‐resistant, obese Zucker fatty rats.^126^ These data accord with the genetic studies suggesting a role for SHIP2 in the development of insulin resistance and DKD.

## 6 SHIP2 MOUSE MODELS SUPPORT A ROLE FOR SHIP2 IN METABOLIC DISORDERS

### 6.1 SHIP2 knockout mouse models

Both long‐ and short‐term mouse models have been utilized to analyse the role of SHIP2 as a regulator of metabolism, including both knockout and overexpression strategies (Table 2). Two different whole‐body knockout mouse models for SHIP2 have been generated. The first model targeted exons 19‐29 of *Inppl1* (mouse SHIP2 gene).^127^ The mice showed increased sensitivity to insulin and reduced expression of hepatic gluconeogenic enzymes, leading to neonatal hypoglycemia and death within 3 days after birth.^127^ Compared to the wild type mice, the adult heterozygous mice showed greater insulin sensitivity, higher expression of GLUT4 at the plasma membrane, and responded to insulin stimulation with higher glycogen synthesis rate in the skeletal muscle. However, the genomic fraction deleted in this model included not only exons 19‐29 of *Inppl1*, but also the third exon of another gene, *Phox2a*.^127^ It therefore remains uncertain whether the phenotype of the mice is caused by absence of *Inppl1*, *Phox2a* or both, or by expression of a truncated form of SHIP2.

**Table 2:** SHIP2 (encoded by *Inppl1*) mouse models and their phenotypes. The mouse model generated by Clément *et al*.^127^ is not included as it remains uncertain whether deletion of *Phox2a* affects the phenotype

SHIP2 *knockout mouse models*			
Model	Generation strategy	Phenotype	Reference
*Inppl1* knockout	Deletion of first 18 exons of *Inppl1*	Developmental defects in skull Reduced body length and weight Normal glucose and insulin levels Normal glucose and insulin tolerance Improved serum lipid values Resistance to high‐fat diet	Sleeman *et al*.^106^
Catalytic inactivation of SHIP2	Deletion of exons 18 and 19 of *Inppl1*	Developmental defects in skull and female genital tract Reduced skeletal muscle weight and adiposity Reduced body weight Normal glucose tolerance and insulin sensitivity Reduced insulin secretion Improved serum lipid values Increased proximal tubule reabsorption	Dubois *et al*.^128^
Catalytic inactivation of SHIP2 in proximal tubules	Deletion of exons 18 and 19 of *Inppl1* in proximal tubules	Increased proximal tubule reabsorption	Sayyed *et al*.^129^
catalytic inactivation of one SHIP2 allele in endothelium	deletion of exons 18 and 19 of *Inppl1* in endothelium	Slightly increased body weight Higher fasting glucose Reduced glucose tolerance and insulin sensitivity Blunted acetylcholine‐ and insulin‐mediated aortic vasodilatation Vascular oxidative stress	Watt *et al*.^130^

SHIP2 *overexpression mouse models*			
Model	Generation strategy	Phenotype	Reference
Overexpression of wild‐type SHIP2 in liver in *db/+* mice	Adenovirus‐mediated gene transfer in the liver, 4 d	Hyperinsulinemia Potentiated increase in plasma glucose after oral glucose intake Systemic insulin resistance	Fukui *et al*.^131^
Overexpression of 5′‐phosphatase defective SHIP2 in liver in *db/db* mice	Adenovirus‐mediated gene transfer in the liver, 4 d	Reduced fasting glucose Improvement of elevated glucose and insulin levels after oral glucose intake	Fukui *et al*.^131^
Overexpression of 5′‐phosphatase defective SHIP2 in liver in KKA^y^ mice	Adenovirus‐mediated gene transfer in the liver, 5 d	Reduced gluconeogenesis Increased hepatic glycogen content Increased glycolysis Increased serum triglycerides Improved glucose tolerance Reduced prandial blood glucose	Grempler *et al*.^132^
SHIP2 transgenic mice	Overexpression of SHIP2 using modified β‐actin promoter	Increased body weight Increased fasting serum insulin Impaired glucose tolerance and insulin sensitivity Reduced hepatic glycogen content at 9 mo	Kagawa *et al*.^133^

The second *Inppl1* knockout strategy deleted the first 18 exons of *Inppl1* including the translation‐initiating ATG, leading to total lack of *Inppl1* mRNA and SHIP2 protein.^106^ These mice lived till adulthood, but showed truncated facial features due to abnormal skeletal growth and reduced body length and weight, despite a tendency of increased food intake. The *Inppl1^−/−^* mice showed normal glucose and insulin levels as well as normal glucose and insulin tolerance, but decreased serum triglycerides, non‐esterified free fatty acids, cholesterol and leptin levels at 12 weeks of age compared to wild‐type littermates. In the basal state, *Inppl1^−/−^* mice showed no difference in the activation of Akt compared to wild‐type mice, but in response to insulin, the null mice showed increased activation of Akt in liver and muscle. Interestingly, the null mice gained less weight when placed on high‐fat diet for 6 weeks, and did not show an increase in serum lipids, insulin or glucose. The *Inppl1* null mice on high‐fat diet also showed a greater basal metabolic rate and increased energy expenditure.^106^ Thus, the lack of *Inppl1* protects the mice against diet‐induced obesity.

A more recent study describes generation of a mouse model with catalytically inactive SHIP2, deleting exons 18 and 19.^128^ These mice had reduced body length and weight, defects in the development of the skull and the female genital tract, and reduced skeletal muscle weight and adiposity. Especially, the males were hyperphagic, their serum triglycerides and cholesterol levels as well as insulin secretion from the mutant islets were lower. No difference was observed in glucose tolerance and insulin sensitivity or signalling,^128^ similar to the *Inppl1* knockout described above.^106^ Further studies on this model revealed increased microvilli formation in the proximal tubules of the kidney due to activation of the ezrin/radixin/moesin family of proteins, leading to increased reabsorption by the proximal tubules.^129^ Specifically, reabsorption of low‐molecular‐weight proteins, glucose and phosphate was increased by the proximal tubule cells. Essentially similar kidney phenotype was observed when *Inppl1* was catalytically inactivated specifically in proximal tubules only.^129^ Interestingly, there was a trend of increased uptake of intravenously injected, fluorescently labelled albumin by proximal tubules after catalytic inactivation of *Inppl1* in proximal tubules.^129^ Watt *et al.* 130 generated mice with catalytic inactivation of one *Inppl1* allele in endothelium‐specific manner to define the role of SHIP2 in vascular function. They found that the mice developed increased vascular oxidative stress and systemic insulin resistance because of impaired glucose uptake in adipose tissue and skeletal muscle. Thus, the study proposes that normal vascular SHIP2 is necessary for maintaining systemic glucose homoeostasis and preventing endothelial dysfunction induced by oxidative stress.^130^

### 6.2 SHIP2 overexpression mouse models

Several studies have addressed the role of SHIP2 in the regulation of metabolism *via* overexpression strategies, inspired by the finding that SHIP2 is upregulated in peripheral insulin sensitive tissues in insulin resistant, diabetic *db/db* mice.^125^, ^126^ Transient, adenovirus‐mediated overexpression of wild‐type SHIP2 in non‐diabetic *db/+* mice led to overexpression of SHIP2 in the liver but not in other tissues.^131^ This decreased insulin‐induced activation of Akt in the liver and increased the expression of gluconeogenic genes, leading to hyperinsulinemia and potentiated the increase in blood glucose after oral glucose intake. This indicates that overexpression of SHIP2 in liver leads to systemic insulin resistance.^131^ Adenovirus‐mediated overexpression of 5′‐phosphatase‐defective SHIP2 (dominant‐negative SHIP2) in diabetic *db/db* mice improved the ability of insulin to induce Akt activation in the liver, and partly reduced the enhanced expression of gluconeogenic genes.^131^ This lowered fasting blood glucose and reduced elevated glucose and insulin levels after oral glucose intake, indicating that inhibition of SHIP2 in liver is effective in ameliorating hyperglycemia. Insulin signalling in skeletal muscle or adipose tissue was not affected in either model.^131^ In another study, mutated form of SHIP2 lacking the 5′‐phosphatase activity was introduced *via* adenoviral infection into KKA^y^ mice, a model of hyperglycemia and hyperinsulinemia.^132^ This resulted in increased basal and insulin‐stimulated Akt activation in liver, decreased glucose production after puryvate challenge, and increased hepatic glycogen content. Hepatic SHIP2 inhibition also increased glycolysis and serum triglycerides, improved glucose tolerance and reduced prandial blood glucose levels after feeding *ad libitum*, supporting the idea that SHIP2 inhibition improves glucose metabolism.^132^

In addition to the above two short‐term models, Kagawa *et al*.^133^ generated transgenic mice overexpressing SHIP2 using a modified β‐actin promoter, leading to SHIP2 overexpression in liver, skeletal muscle, white adipose tissue, pancreas, brown adipose tissue and brain. The transgenic mice gained more body weight and showed increased fasting plasma insulin, whereas fasting glucose, leptin, adiponectin, total cholesterol, triglycerides and non‐esterified fatty acids did not differ from the wild‐type littermates under normal diet. However, the transgenic mice showed impaired glucose tolerance and insulin sensitivity. This could be caused by reduced activity of the PI3K pathway, as insulin‐stimulated activation of Akt was reduced in adipose tissue, skeletal muscle and liver in the SHIP2 overexpressing mice. The expression of gluconeogenic genes in the liver was increased and glycogen content decreased. These data indicate that increased expression of SHIP2 impairs glucose metabolism and insulin sensitivity.^133^

## 7 SHIP2 INHIBITORS AMELIORATE METABOLIC DISORDERS

The in vitro and in vivo studies described above, revealing that SHIP2 overexpression suppresses insulin signalling and debilitates glucose metabolism, have increased the interest to develop SHIP2 inhibitors as a potential therapeutic approach to treat obesity‐induced insulin resistance and T2D. A number of inhibitors have been identified, but for most of them, no data on metabolic effects are available and some inhibit also SHIP1 (see discussion on the beneficial metabolic effects of SHIP1 inhibition in paragraph 10).^134^, ^135^, ^136^, ^137^, ^138^ A few studies describe the effects of SHIP2 inhibition on metabolic parameters either in cells in vitro or in vivo.^139^, ^140^, ^141^, ^142^ Interestingly, we recently identified metformin, the first‐line medication used to treat T2D, as a novel SHIP2 inhibitor.^140^ The following section focuses on the SHIP2 inhibitors whose roles in metabolic disorders have been defined (Figure 3). The metabolically less well‐characterized SHIP2 or SHIP1/SHIP2 inhibitors are summarized in recent reviews.^108^, ^119^

### 7.1 Small molecule SHIP2 inhibitors

Using malachite green phosphate assay for measuring SHIP2 activity, Suwa *et al*.^142^ screened in a high‐throughput approach a chemical library for inhibitors of the phosphatase domain of SHIP2. This led to the identification of AS1949490, which inhibited human SHIP2 with an IC_50_ value of 0.62 µmol/L and a Ki value of 0.44 µmol/L, but did not inhibit other phosphatases, such as SHIP1, PTEN, synaptojanin or myotubularin. In L6 myotubes, AS1949490 increased insulin‐induced activation of Akt in a dose‐dependent manner, and increased both glucose consumption and glucose uptake. In FAO hepatoma cells, AS1949490 reduced glucose production, and acute administration of the inhibitor to normal mice reduced the expression of gluconeogenic genes in the liver. Furthermore, administration of AS1949490 to *db/db* mice for 7‐10 days reduced both fasting and non‐fasting blood glucose and reduced the area under the curve in oral glucose tolerance test. This was accompanied by increased phosphorylation of GSK3β in the liver, an indication of the activation of insulin signalling.^142^ These findings supported the idea that inhibition of SHIP2 has therapeutic potential as a treatment for T2D.

The same group further identified another novel SHIP2 inhibitor, AS1938909, with an IC_50_ value of 0.57 µmol/L and a Ki value of 0.44 µmol/L for human SHIP2.^141^ This inhibitor was also highly selective for SHIP2, and increased Akt activation, glucose consumption and glucose uptake in L6 myotubes. Both AS1938909 and the previously identified AS1949490 increased the expression level of GLUT1 mRNA in L6 myotubes, which could contribute to the increased glucose uptake.^141^ The effects of AS1938909 in vivo were not defined.

Ichihara *et al*.^142^ utilized in silico ligand‐based drug design combining the structures of AS1949490 and NGD‐61338, another novel SHIP2 inhibitor with an IC_50_ value of 1.1 µmol/L,^134^ to design new SHIP2 inhibitor candidates.^139^ The designed compounds and their derivatives were tested for their ability to activate Akt as an indirect measure of SHIP2 inhibition. Compound N‐[4‐(4‐chlorobenzyloxy)pyridine‐2‐yl]‐2‐(2,6‐difluorophenyl)‐acetamide (CPDA), the most potent of these compounds, was further tested in *db/db* mice. Treatment of the mice with CPDA for 10‐13 days decreased the fasting blood glucose, reduced hepatic expression of gluconeogenic genes and ameliorated glucose intolerance, and the effects were comparable to those of AS1949490.^139^

### 7.2 Antidiabetic drug metformin inhibits SHIP2

We utilized in silico structure‐based virtual screening of small molecule chemical libraries to identify SHIP2 inhibitors, and one of the identified molecules was metformin.^140^ This supports the proposition that metformin not only acts *via* reducing gluconeogenesis in the liver, but also enhances insulin sensitivity and glucose uptake in peripheral tissues. The early studies on the mechanisms of action of metformin revealed that mitochondria are central for the effects of metformin in liver.^143^, ^144^ To produce ATP, mitochondria carry out oxidation of NADH, produced by glycolysis or β‐oxidation of fatty acids, generating NAD^+^ and electrons. The electrons are transferred through the respiratory chain complexes I, II, III and IV to O_2_, eventually generating H_2_O. The protons generated by oxidation of NADH are pumped to the intermitochondrial membrane through respiratory complexes I, III and IV, and the proton gradient drives ATP synthase to generate ATP from ADP. Metformin has been shown to inhibit the complex I (NADH:ubiquinone oxidoreductase) leading to reduced cellular respiration.^143^, ^144^ The reduction in cellular ATP concentration and increase in ADP/ATP and AMP/ATP ratios activates the energy sensor AMP‐activated protein kinase (AMPK). Indeed, Zhou *et al*.^145^ revealed that metformin activates AMPK and thereby suppresses hepatic glucose production and expression of lipogenic enzymes, induces fatty acid oxidation, and also enhances glucose transport to muscle, leading to reduced plasma glucose and triglycerides. Metformin also upregulates PPARγ coactivator 1⍺ (PGC‐1⍺) *via* AMPK, and selectively downregulates transcription factors that facilitate PGC‐1⍺‐mediated gluconeogenesis, thus suppressing gluconeogenesis.^146^ Metformin can affect hepatic metabolism also *via* AMPK‐independent pathways, as metformin reduced gluconeogenesis in mice deficient of AMPK, leading to the proposition that metformin inhibits hepatic gluconeogenesis *via* decreased energy state.^147^ Notably, the effects of metformin on AMPK activity are apparently concentration dependent, as low concentrations of metformin, as detected in the portal vein, suppressed gluconeogenesis *via* activation of AMPK, without increasing the cellular AMP/ATP ratio.^148^

Metformin has also been shown to induce the expression of fibroblast growth factor 21 (FGF21), an endocrine hormone with anti‐obesity and anti‐diabetes properties, in the liver in AMPK‐independent manner by inhibiting mitochondrial complex I.^149^ Also, metformin‐induced accumulation of AMP abrogates the glucagon‐induced activation of adenylate cyclase.^150^ This leads to reduced level of cyclic AMP (cAMP) and reduced activation of protein kinase A (PKA) and its targets, and, thus, inhibition of gluconeogenesis.^150^ In addition to affecting mitochondrial complex I, metformin has been shown to directly bind to the redox shuttle enzyme mitochondrial glycerophosphate dehydrogenase.^151^ Inhibition of this enzyme by metformin leads to altered hepatic redox state, reduction of the conversion of lactate and glycerol to glucose, and reduced hepatic gluconeogenesis.^151^ However, the finding that metformin causes an acute inhibition of glycerophosphate dehydrogenase has recently been challenged.^152^ In addition, metformin exerts some of its beneficial metabolic effects *via* altering the gut microbiota by affecting genes encoding metalloproteins or metal transporters,^153^ another facet of the multiple modes of action of the drug.

As introduced above, in the liver the only direct target of metformin is glycerophosphate dehydrogenase,^151^ although this has been challenged.^152^ SHIP2 represents the second direct molecular target identified for metformin.^140^ In in vitro experiments with purified recombinant proteins, metformin was found to directly bind to and inhibit the catalytic activity of the recombinant SHIP2 phosphatase domain with an IC_50_ value of 6.0 µmol/L, but it did not inhibit SHIP1 or PTEN confirming the specificity of metformin for SHIP2.^140^ Metformin also inhibited the activity of SHIP2 in cultured L6 myotubes and podocytes and increased glucose uptake in both cell types. In L6 myotubes, metformin increased the amount of GLUT4 at the plasma membrane by inhibiting endocytosis of GLUT4 and thereby enhanced glucose uptake, without affecting Akt activity. In vivo, metformin inhibited SHIP2 activity in muscle and kidney tissue of *db/db* mice after 12 days of treatment. This short‐term metformin treatment enhanced insulin sensitivity of the *db/db* mice, visualized by reduced glucose area under the curve in insulin tolerance test, but hyperglycemia was not reduced. This could be due to the compensatory gluconeogenesis in the kidney.^140^ Even though the major site of metformin action has previously shown to be inhibition of gluconeogenesis in the liver,^154^ and the 12‐day metformin treatment reduced the expression of gluconeogenesis genes in the liver, metformin did not inhibit the activity of SHIP2 in cultured hepatoma cells or in the liver of *db/db* mice.^140^ It thus appears that metformin has tissue‐specific direct targets, mitochondrial glycerophosphate dehydrogenase in the liver^151^ (see, however^152^) and SHIP2 in muscle and kidney tissue.^140^ In addition to direct binding to SHIP2, various other indirectly activated pathways have been shown to be involved in metformin‐induced glucose uptake in muscle tissue and as in liver, they occur *via* both AMPK‐dependent and ‐independent mechanisms. For example, metformin has been shown to activate atypical protein kinase C (aPKC) *via* AMPK‐, ERK‐ and PDK1‐dependent pathway to enhance glucose uptake in muscle.^155^ A later study revealed that metformin increases glucose uptake independent of AMPK *via* novel and conventional PKC isoforms.^156^ Metformin has also been shown to increase the abundance of GLUT1 at the plasma membrane^157^ and to decrease GLUT4 endocytosis in muscle cells.^140^, ^158^

One of the challenges in metformin research has been the great variability in experimental concentrations and treatment times, making comparisons between studies difficult. Of note, cells in culture tend to lose the receptors *via* which metformin enters the cells.^159^ Therefore, the concentrations of metformin required in the in vitro experiments may greatly vary depending on the cell type, whether the line is a primary cell line or an immortalized cell line maintained in culture for a long period of time, and on the exposure time to metformin.

Collectively, several SHIP2 inhibitors have been identified but only four of them have been analysed in vitro or in vivo and shown to improve metabolic parameters (Figure 3). Identification of metformin as a SHIP2 inhibitor and the findings described above, indicating that SHIP2 inhibition improves glucose metabolism, indicate that SHIP2 is an excellent target to design new treatments to ameliorate insulin resistance and T2D.

## 8 DOES SHIP2 CONVEY THE RENOPROTECTIVE EFFECTS OF METFORMIN?

In addition to its major action in reducing hyperglycaemia, metformin has been shown to have renoprotective effects: It reduces albuminuria in patients with T2D^160^, ^161^ and in a rat model of T2D,^162^ and also increases the estimated glomerular filtration rate in patients with T1D.^163^ Various mechanisms have been proposed. In rats with T2D, metformin increases the expression of nephrin^164^ and podocalyxin,^165^ both known to be downregulated or mislocalized in DKD.^21^, ^22^, ^23^, ^24^, ^164^, ^165^ Treatment of primary rat podocytes with metformin activates protein deacetylase sirtuin 1 (SIRT1) and AMPK, and prevents high glucose‐induced downregulation of SIRT1 expression.^166^ Metformin also reduces high glucose‐induced apoptosis of podocytes in vitro.^167^ Interestingly, podocyte loss was found to be more pronounced in patients with T2D receiving non‐metformin medication as compared to patients receiving metformin or people without diabetes, supporting a renoprotective role for metformin.^140^ This, coupled to the finding that SHIP2 activity is elevated in the kidneys of patients with T2D receiving other than metformin medication proposes that metformin could protect podocytes from injury *via* inhibiting SHIP2 activity.^140^ This proposition was supported by in vitro studies on immortalized human podocytes, in which SHIP2 overexpression has been shown to enhance apoptosis coupled with reduced Akt activation.^126^ Metformin restored SHIP2 overexpression‐induced reduction in Akt activation and normalized the level of apoptosis.^140^ An earlier study revealed that upregulation of SHIP2 in podocytes in diabetic animal models occurred already before the reported age of onset of proteinuria, and before any histological changes in the glomeruli were observed.^126^ This indicates that upregulation of SHIP2 is not a secondary effect of podocyte damage but rather contributes to it, and raises an intriguing question whether SHIP2 inhibition at an early stage of diabetes could prevent podocyte injury and development of DKD. Answering this question calls for carrying out a randomized controlled trial with metformin.

The above examples and the previous paragraph illustrate the great complexity of the mechanisms of action of metformin. Clearly, more work is necessary to understand the exact molecular mechanisms *via* which metformin acts in different tissues, and specifically, to define the central pathways *via* which metformin acts in patients with T2D in long‐term treatments. A great amount of work needs to be carried out with the new SHIP2 inhibitors to define their exact mechanisms of action in different tissues, and to identify the potential SHIP2‐independent mechanisms of action. This will aid in the drug development process when designing derivatives of the best candidate molecules to further improve their characteristics. The mechanistic understanding of how the inhibitor works will also help in identifying the potential adverse effects.

## 9 BENEFITS AND CAVEATS OF INHIBITING SHIP2

As described in the above paragraph, the effects of SHIP2 inhibition on metabolic parameters in vitro and in vivo point out a clear beneficial role for SHIP2 inhibition in reducing insulin resistance and hyperglycemia and improving glucose metabolism.^139^, ^140^, ^141^, ^142^ In addition, SHIP2 inhibition by metformin protects podocytes from injury at a stage when SHIP2 expression or activity is elevated, such as in DKD,^140^ proposing that SHIP2 inhibition could be a potent approach to prevent DKD. This is a fascinating aspect as the subgroup of T2D‐patients with severe insulin resistance is more susceptible to develop DKD,^70^ raising a question whether intensive treatment of these patients with metformin, or in future, with more potent SHIP2 inhibitors, could provide an effective means to prevent the development and progression of this fierce complication. Further studies are also needed to define whether the renoprotective effects of SHIP2 inhibition are due to reduction of general peripheral insulin resistance or direct protective effects of SHIP2 inhibition on podocytes.

The genetic mouse models to a large part support a protective role for reduced expression or lowered catalytic activity of SHIP2 against metabolic disorders, with the exception of catalytic inactivation of one *Inppl1* allele in vasculature.^130^ This led to increased oxidative stress, endothelial dysfunction and systemic insulin resistance, and the authors raised a concern for SHIP2 inhibition in endothelium.^130^ Previously, no such effects were described in the whole body *Inppl1* knockout,^106^ and Watt *et al*.^168^ do not speculate what could be the reason for such a difference in the metabolic outcome in these two models. The studies with SHIP2 inhibitors, described above, do not raise concerns on systemic insulin resistance but rather report contrary findings. Furthermore, expression of catalytically inactive SHIP2 in hepatic HepG2 cells has been shown to reduce ROS generation. Also, inhibition of SHIP2 with AS1949490 in CD2AP‐deficient podocytes, showing increased oxidative stress, reduced ROS generation.^169^ Despite this, increased apoptosis, caused by lack of CD2AP, was not reduced but aggravated by SHIP2 inhibition, indicating that when CD2AP is missing, SHIP2 inhibition may have unexpected consequences.^169^ Uncovering the mechanisms involved calls for further studies using in vivo models. The studies in cultured cells or short‐term administration of SHIP2 inhibitors in vivo may not reflect the outcome of long‐term inhibitor administration, and there may also be cell‐type specific differences in the effects of SHIP2 inhibition. Also, the uptake of the various inhibitors to different cell types very likely varies, which may result in distinct outcomes compared to genetic inactivation of SHIP2. SHIP2 has also functions that are independent of its catalytic activity, relying on its protein‐protein interactions.^118^ It may thus be that in certain cases the genetic knockout models produce a phenotype that does not directly reflect the outcome of reducing only the catalytic activity of SHIP2 with inhibitors, leaving the protein interaction domains still functional.

An interesting beneficial aspect of the catalytic inactivation of SHIP2 in mice, either generally or specifically in kidney proximal tubules, is the increased reabsorption ability of the proximal tubules.^129^ The study showed both enhanced reabsorption of low‐molecular‐weight proteins as well as a trend of increased uptake of injected albumin.^129^ The authors proposed that SHIP2 could represent a new therapeutic target for renal Fanconi syndrome, characterized by urinary loss of low molecular weight proteins and metabolites due to either acquired or inherited dysfunction of proximal tubules.^129^ The authors were able to mimic some of the aspects of the genetic model with a SHIP2 inhibitor in cultured cells in vitro,^129^ but the effects of SHIP2 inhibition with small molecule inhibitors in vivo on proximal tubule structure and functional properties await further studies. It will also be interesting to define whether SHIP2 inhibition in glomerular diseases presenting with increased leakage of albumin, such as DKD, show increased tubular uptake of albumin achieved by SHIP2 inhibition.

SHIP2 inhibition has also been addressed in the field of cancer research as a potential treatment for specific types of cancer. This is a reflection of a paradigm shift in the cancer field as it comes to phosphatases, including SHIP2.^135^ Phosphatases were earlier considered rather as tumour suppressors, but the finding that they can activate signalling pathways proposes that they could actually be potential oncogenes and thereby targets for cancer treatment. In the case of SHIP2, its hydrolysis product, PtdIns(3,4)P2, binds to Akt more potently than PtdIns(3,4,5)P3.^170^ Furthermore, both PtdIns(3,4)P2 and PtdIns(3,4,5)P3 are required for the high‐level activation of Akt.^171^ It has been proposed that both PtdIns(3,4)P2 and PtdIns(3,4,5)P3 are necessary for cancer cell survival and malignancy, and both their absolute amount and ratio control cell death.^172^ In line with this “two PIP hypothesis”, studies revealing SHIP2 as a target to treat cancer and showing beneficial effects of SHIP2 inhibitors are emerging. In clinical specimens of breast,^173^ colorectal,^136^, ^174^ non‐small cell lung^175^ and hepatocellular^176^ cancer, the expression level of SHIP2 has been shown to be elevated and to correlate with decreased patient survival. Beneficial effects of SHIP2 inhibition have been proposed in breast^135^, ^177^ and colorectal cancer.^136^ In gastric cancer specimens, however, SHIP2 expression is frequently downregulated, and this enhances the malignant behaviour of gastric cancer cells.^178^ Accordingly, it appears that the effects of SHIP2 in different cells are context dependent. It is important to note, that mice that express catalytically inactive SHIP2 survive longer than 18 months and show no signs of tumour development,^128^ and neither do mice depleted of SHIP2.^106^ These data increase the prospects of SHIP2 as a potential cancer treatment target in specific types of cancer in which SHIP2 is overexpressed or overactivated. The benefits and caveats of SHIP2 inhibition are summarized in Table 3.

**Table 3:** Benefits and caveats of inhibiting the catalytic activity of SHIP2. See the text for details and references

Benefits	Caveats
General metabolism	
Reduction of insulin resistance and hyperglycemia Improvement of glucose metabolism	Genetic SHIP2 inhibition in endothelium leads to systemic insulin resistance Many SHIP2 inhibitors lack studies of their effects after long‐term administration in vivo
Kidney	
Protection of podocytes from SHIP2 overexpression ‐induced apoptosis in vitro Reduced podocyte loss in patients with T2D receiving metformin compared to patients receiving other medication Increased solute reabsorption by proximal tubules	Increased apoptosis of SHIP2 inhibitor ‐treated CD2AP‐deficient podocytes in vitro
Cancer	
Potential for treatment of specific types of cancer No reports on cancer development in knockout mice or mice expressing catalytically inactive SHIP2	Effects in different cell types are context dependent

## 10 FUTURE PROSPECTS

The SHIP2 inhibition studies carried out thus far with AS1949490 and CPDA in vivo in mice have covered relatively short‐term treatments, remaining shorter than 2 weeks.^139^, ^142^ The long‐term use of SHIP2 inhibitor metformin is safe, except that metformin is contraindicated when kidney function is declining. Long‐term treatments of diabetic rodent models with AS1949490, CPDA and other new SHIP2 inhibitors described above are warranted to confirm that these inhibitors per se do not cause harmful side effects. New SHIP2 inhibitors with better drug‐like characteristics (for example, solubility and bioavailability) than those of the previously characterized SHIP2 inhibitors would be welcome as leads for drug development. The recent classification of patients with diabetes to five distinct groups with specific profiles of disease progression and risks for complications^70^ offers, for the first time, possibilities for precision medicine in diabetes. In terms of the kidney complication, the patients who are severely insulin resistant are at high risk to develop DKD.^70^ The potential of SHIP2 inhibitors to act as insulin sensitizers highlights their potential to provide new leads for personalized medications for this subgroup of patients.

Some of the developed inhibitors are panSHIP inhibitors that have been tested in cancer cells,^135^ but their effects on metabolic disorders are thus far uncharacterized. At least inhibition of SHIP1 with a SHIP1‐specific inhibitor K118 can be highly beneficial, as K118 improves the metabolic parameters of diet‐induced obese mice by attenuating inflammation in the visceral adipose tissue.^179^ The K118 treatment was relatively short, 4 weeks; a longer treatment would be needed to confirm that no side effects occur upon time. This, and treatment of diabetic rodent models with either panSHIP inhibitors or with a combination of SHIP1 and SHIP2 inhibitors will be needed to define the possible superiority of the combined inhibition over inhibition of each protein alone.

The finding that patients with T2D receiving metformin exhibit less podocyte loss than patients with other medication^140^ raises several fundamental research lines. To show that metformin acts renoprotectively in patients with T2D, a randomized controlled trial is needed. The other research line to pursue is the identification of SHIP2 inhibitors more potent than metformin, especially in terms of better inhibition of SHIP2 in podocytes, followed by defining whether these molecules provide better renoprotection than metformin in diabetic animal models with kidney disease. One could envisage that highly insulin resistant patients at high risk to develop DKD^70^ would greatly benefit from treatments targeting SHIP2 with an inhibitor enhancing insulin sensitivity as well as protecting kidneys from injury. Furthermore, knowing that SHIP2 is upregulated in several types of cancer and that this correlates with decreased patient survival, raises an intriguing question whether SHIP2 inhibitors could provide potential means also for new personalized treatment options for cancer patients.

## CONFLICTS OF INTEREST

The author declares no conflicts of interest.
